# Development and initial validation of a short form of the Memories of Home and Family Scale

**DOI:** 10.1007/s42844-023-00097-x

**Published:** 2023-04-29

**Authors:** Enya Redican, Caitlyn Rawers, Eoin McElroy, Philip Hyland, Thanos Karatzias, Menachem Ben-Ezra, Mark Shevlin

**Affiliations:** 1 Ulster University, School of Psychology, Cromore Road, Coleraine, Co, Londonderry, BT52 1SA UK; 2 Department of Psychology, Maynooth University, Kildare, Ireland; 3 Edinburgh Napier University, School of Health and Social Care, Edinburgh, UK; 4 School of Social Work, Ariel University, Ariel, Israel

**Keywords:** Reliability, Validity, Confirmatory factor analysis, Exploratory factor analysis

## Abstract

The Memories of Home and Family Scale (MHFS; Shevlin et al., 2022) was developed as a multidimensional measure of subjective memories of experiences at home and with family during childhood. Due to the length of the scale, a short version of the MHFS (MHFS-SF) has been developed. Data were from Wave 7 of the COVID-19 Psychological Research Consortium Study (C19PRC-UK), a population based UK survey (*N* = 1405). Two items with the highest factor loadings from each of the six dimensions of the original MHFS were selected for inclusion. Confirmatory factor analytic (CFA) models were estimated to test the dimensionality of the scale. Convergent and discriminant validity were tested by examining associations with criterion variables. CFA results supported the multidimensionality of the scale. MHFS-SF total and sub-scale scores were negatively correlated with measures of depression, anxiety, loneliness, and paranoia, and were positively correlated with wellbeing. Regression analyses revealed that MHFS-SF total and sub-scale scores significantly predicted loneliness, paranoia, and wellbeing, even after accounting for age, gender, and current internalising symptoms. Results from this study suggest that the MHFS-SF scores retain the excellent psychometric properties of the original scale while improving efficiency. The MHFS-SF demonstrated high levels of convergent and discriminant validity with mental health and wellbeing measures. Future research should seek to validate the MHFS-SF in different populations and assess its usefulness in clinical settings.

**Supplementary Information:**

The online version contains supplementary material available at 10.1007/s42844-023-00097-x.

It is widely acknowledged that adverse childhood experiences (ACEs) including abuse, neglect, and household dysfunction can have profound and lifelong impacts on physical health and psychological wellbeing (e.g., Campbell et al., 2016; Felitti et al., 1998; Hughes et al., 2017; Kalmakis and Chandler, 2015). Despite ACEs being highly prevalent (e.g., Bellis et al., 2019; Merrick et al., 2018), research shows how individuals affected by ACEs often demonstrate patterns of healthy and adaptive functioning, or resilience (Bonanno, 2004). Benevolent childhood experiences (BCEs), positive experiences occurring during the first eighteen years of life that promote perceptions of love, comfort, safety, security, and consistency have been proposed as a potential contributing factor for this capacity for resilience in the face of adversity (Narayan et al., 2018). Emerging evidence suggests that higher levels of BCEs are associated with better health and psychological wellbeing, even for individuals affected by ACEs (e.g., Almeida et al., 2021; Doom et al., 2021; Zhan et al., 2021; Crandall et al., 2019, 2020; Karatzias et al., 2020; Narayan et al., 2018; Merrick et al., 2019; Bethell et al., 2019). Findings surrounding BCEs largely mirror the well-established literature on angels in the nursery, which describe benevolent memories of feeling loved, safe, and protected during childhood (Narayan et al., 2020). Research has consistently demonstrated how such memories can protect against the pervasive effects of childhood maltreatment on psychological wellbeing (e.g., Narayan et al., 2007).

There are numerous measures available which assess positive early life experiences including the BCE scale (Narayan et al., 2018), the seven-item Positive Childhood Experiences (PCE) Score (Bethell et al., 2019) and the eight-item Childhood Caregiving Environment Scale (CCE scale; Abbott & Slack, 2021). There are several gaps within these existing measures such as the use of dichotomous response options, the focus on objective recollections only and the narrow range of experiences examined (Shevlin et al., 2022). Other measures focused on positive familial experiences include the Family Environment Scale (FES; Moos & Moos, 1996), the Parental Acceptance Questionnaire (PAQ; Rohner et al., 2005), and the Experiences in Close Relationships- Relationship Structures (ECR-RS) scale (Fraley et al., 2011). Limitations of these particular measures include that the FES uses a dichotomous response format while the PAQ and ECR-RS are focused on relationships with individual family members. Limitations of existing measures necessitated a novel measure of BCEs; hence the development of the Memories of Home and Family Scale (MHFS; Shevlin et al., 2022).

Developed as a positive analogue to the short-form version of the Childhood Trauma Questionnaire (Bernstein et al., 2003), the MHFS is a self-report measure which captures recollections of experiences at home and family during early development. The MHFS is comprised of 28 items organized across six dimensions including (i) being a valued member of the family, (ii) being an independent member of the family, (iii) feeling supported within the family, (iv) feeling secure within the family, (v) a sense of wellbeing at home, and (vi) experiences of growth and meaning. As highlighted by Shevlin et al. (2022), the MHFS adds to existing measures in several ways. First, the multi-category response scale (as compared to the dichotomous response options provided in the BCE scale and PCE score) facilitates the exploration of the role of frequency of positive experiences in determining health and psychological wellbeing outcomes. Second, guided by research indicating subjective recollections of childhood experiences to be powerful predictors of later outcomes (e.g., Richter et al., 2009), the MHFS focuses exclusively on subjective memories of positive emotions and experiences during childhood (as compared to objective recollections in the BCE scale and PCE score). Third, the MHFS includes a wide array of BCEs and provides both subscale and total scores (as compared to both the narrow range of items and the provision of total scores only in existing measures). Finally, the exclusive focus on a wide spectrum of BCEs pertaining to the family and home environment is beneficial given that a positive family environment plays a powerful role in shaping a young person’s development and overall wellbeing (Xie et al., 2022).

In the initial validation study, the MHFS performed well as evidenced by the high levels of internal reliability and convergent validity (Shevlin et al., 2022). However, the length of the scale may be burdensome for respondents and hinder the inclusion of the MHFS in surveys and clinical practice. Consequently, the current study sought to construct and validate a short form version of the MHFS (MHFS-SF). Given that one of the unique features of the MHFS is its multidimensional structure and the inclusion of a broad array of BCEs within the family caregiving context, it was necessary to ensure that the short form version retained these aspects. Hence, based on the CFA results from the initial validation study (Shevlin et al., 2022), the two items with the highest factor loadings from each of the six subscales were selected for the short form version. The first aim of the present study was to determine the latent structure of the MHFS-SF. Similar to the original MHFS, it was anticipated that the MHFS-SF would capture a multidimensional construct. The second aim of the current study was to examine the internal reliability and convergent validity of the MHFS-SF. Consistent with the initial validation study (Shevlin et al., 2022), it was hypothesized that the MHFS-SF would possess high levels of internal reliability and that total and sub-scale MHFS-SF scores would be negatively correlated with depression, anxiety, and loneliness. Moreover, it was hypothesized that total and sub-scale MHFS-SF scores would be positively associated with wellbeing. The final aim of the current study was to establish both predictors of total and sub-scale MHFS-SF scores and the ability of the total and sub-scale MHFS-SF scores to predict a range of mental health outcomes.

## Methods

### Participants

Data for the current study was derived from Wave 7 of the COVID‐19 Psychology Research Consortium (C19PRC) Study. The C19PRC was designed to assess the psychological, social, and economic effect of the COVID-19 pandemic in different populations (McBride et al., 2021). Procedural details of the C19PRC study have been discussed extensively elsewhere (McBride et al., 2020). Participants for Wave 7 included 1405 adults aged 18 to 89 (*M* = 48.8 years, *SD* = 15.1 years). Participants had either previously answered surveys as part of earlier waves in the C19PRC Study and were recontacted to participate in Wave 7 or were contacted for participation as part of a sample replenishment procedure for Wave 7. All surveys were completed through the online survey company Qualtrics. Because the MHFS-SF items were only included in Wave 7 of the C19PRC study, any participants who withdrew from the C19PRC or who had failed to complete the Wave 7 survey were excluded. The MHFS-SF items were included in Wave 7 of the C19PRC study to identify some potential protective factors for the mental health implications of the COVID-19 pandemic. Of the 1405 participants, 49.8% were male, 50.0% were female, and 0.2% were another category (transgender or prefer not to say). Gender was dummy coded for analysis with males as the reference category.

### Measures

#### Memories of Home and Family Scale – Short Form (MHFS-SF)

Two items with the highest factor loadings were selected from each of the six subscales of the original 28-item scale to comprise the 12-item short form version. The original subscales covered the domains of being a valued member of the family (e.g., “I felt my parents valued me”), being an independent member of the family (e.g., “My family listened to me”), feeling supported (e.g., “My family were supportive”), feeling secure (e.g., “I felt secure at home”), having a sense of wellbeing at home (e.g., “I was happy at home”), and opportunities for growth and meaning (e.g., “My family supported me in reaching my goals”). Participants were asked to recall their early life experiences at home and with their family up to the age of 16 years and rated each item on a five-point Likert scale ranging from ‘Never’ (1) to ‘Always’ (5). Preliminary analysis of the original 28-item scale suggested good convergent validity and reliability (Shevlin et al., 2022).

#### Depression

The Patient Health Questionnaire – 9 (PHQ-9; Kroenke et al., 2001; Spitzer et al., 1999) asks participants to rate nine symptoms of Major Depressive Disorder on the basis of severity in the past two weeks. Participants ranked items such as “Little interest or pleasure in doing things” on a four-point Likert scale ranging from “Not” at all (0) to “Nearly” every day (3). The PHQ-9 has demonstrated good reliability and criterion validity in several populations (Levis et al., 2019). Items were summed to create a total score of depressive symptoms. The internal reliability of the PHQ-9 in this study was excellent (*α* = 0.93).

#### Anxiety

The Generalized Anxiety Disorder (GAD-7; Spitzer et al, 2006) scale measures severity of seven anxiety symptoms, for example “Feeling nervous, anxious or on edge” over the past two weeks. Participants ranked each item using a four-point Likert scale from “Not at all” (0) to “Nearly every day” (3). The GAD-7 has displayed good convergent and discriminant validity and internal consistency (Johnson et al., 2019; Kroenke et al., 2007; Swinson, 2006). Items were summed to create a total score of anxiety symptoms. The internal reliability of the GAD-7 in this study was excellent (*α* = 0.95).

#### Loneliness

A three-item short form version of the Revised UCLA Loneliness Scale (Hughes et al., 2004) assessed general feelings of loneliness on a three-point Likert scale ranging from “Hardly ever” (1) to “Often” (3). Items include lacking companionship, feeling left out, and feeling isolated. The scale has been shown to have acceptable reliability and validity (Hughes et al., 2004). Items were summed to create a total score, with higher scores indicating greater feelings of loneliness. The internal reliability of the loneliness scale in this study was excellent (*α* = 0.90).

#### Paranoia

Paranoid thoughts were assessed using the short-form version of the Persecution and Deservedness Scale (PaDS; Elahi et al., 2017; Melo et al., 2009). The original scale contains two 10-item subscales measuring severity of persecutory beliefs and deservedness of persecution. Five items from the persecution subscale were used to assess the severity of paranoid thoughts, for example “*I’m often suspicious of other people’s intentions towards me”*. Items were rated on a five-point Likert scale from “Strongly disagree” (1) to “Strongly agree” (5). The original and short-form PaDS have demonstrated good internal consistency and convergent validity in clinical and non-clinical samples (Elahi et al., 2017; Melo et al., 2009). Items were summed, with higher scores indicating greater severity of paranoid thinking. The internal reliability of the short-form version of the scale was good (*α* = 0.88).

#### Mental Wellbeing

The Short Warwick-Edinburgh Mental Wellbeing Scale (SWEMWS; Stewart-Brown et al., 2009) is a seven item version of the original 14-item scale that assesses general mental wellbeing over the past two weeks on a five-point Likert scale from “None of the time” (1) to “All of the time” (5). The SWEMWS has shown good construct validity across different populations (Ng Fat et al., 2017; Shah et al., 2021). Items were summed for a total wellbeing score, with higher scores indicating greater mental wellbeing. The internal reliability of the SWEMWS was excellent (*α* = 0.93).

See Supplementary Materials 1 for descriptive statistics of the mental health and wellbeing scales.

## Data Analytic Procedure

Three confirmatory factor analysis (CFA) models were estimated concordant to the original scale (Shevlin et al., 2022): a single factor model, a six factor first-order model, and a one factor second-order model (six first-order latent factors loaded onto a single latent second-order variable representing ‘positive experiences’).The data were deemed suitable for factor analysis as Bartlett’s Test of Sphericity was significant (*p* < 0.001) and the Kaiser–Meyer–Olkin Measures of Sampling Adequacy was 0.975, above the recommended value of 0.50 (Watkins, 2018). Models were tested using MLR estimation (Yuan & Bentler, 2000) in Mplus 8.0 (Muthén & Muthén, 2017).

Model fit was assessed using the chi-square statistic, the comparative fit index (CFI; Bentler, 1990), the Tucker-Lewis index (TLI; Tucker & Lewis, 1973), the root mean square error of approximation (RMSEA; Browne & Cudeck, 1992) and the Standardized Root Mean Square Residual (SRMR; Jöreskog & Sörbom, 1981). Conventional criteria for good model fit include a non-significant chi-square value (*p* > 0.05), CFI and TLI values ≥ 0.90 for adequate fit, RMSEA values < 0.08 for adequate fit, and SRMR values ≤ 0.08 for good fit (Hu & Bentler, 1999).

Next, bivariate correlations were calculated between the best fitting CFA models of the MHFS-SF and age, total scores of depression (measured by PHQ-9), anxiety (measured by GAD-7), loneliness (measured by UCLA Loneliness Scale), paranoia (measured by PaDS) and wellbeing (measured by SWEMWS). Cohen’s (1988) guidelines were followed to interpret the magnitude of the associations (< 0.30 = small, 0.30-0.50 = moderate, > 0.50 = large). Finally, multivariate regressions were estimated predicting mental health and wellbeing outcomes (loneliness, paranoia, and wellbeing) from age, gender, depression, anxiety, and MHFS-SF total scores to test the convergent validity of the MHFS-SF. Additionally, regression models predicting mental health and wellbeing outcomes (loneliness, paranoia, and wellbeing) from age, gender, depression, anxiety, and MHSF-SF subscales were conducted. It should be noted that to determine the unique influence of each of the subscales, these regression models were estimated without adjusting for scores on all other subscales. There was no evidence of multicollinearity as all tolerance values were above 0.2 (minimum = 0.59), and all VIF were below 4 (maximum = 1.69; Garson, 2012). Analysis of Cook’s distance scores found no values above 0.25, below the recommended cut-off of 1, suggesting no influential multivariate outliers were influencing the data (Garson, 2012). However, examination of residuals suggested heteroscedasticity and non-normality. The large sample size of this study precluded the need to transform the data to reduce non-normality (Pek et al., 2017); however, MLR estimation uses robust standard errors, which can account for heteroscedasticity (Muthén & Muthén, 2017).

## Results

### Factor Analysis

Item level statistics, including skew and kurtosis information, are reported in Table 1. The range of possible and observed scores for the short form scale was 12 to 60, this indicates that the scale can generate sufficient variability in scores. The average score was 45.3 (*SD* = 12.3). Internal consistency for all the items, as measured by Cronbach’s alpha, was excellent (α = 0.98).

**Table 1:** Item level Descriptive Statistics for the 12-item Memories of Childhood and Family Scale

Item	Mean	*SD*	Item-total correlation	Skew(SE)	Kurtosis (SE)
1. I felt my parents valued me	3.74	1.11	.91*	-.54 (.07)	-.43 (.13)
2. I felt appreciated by my family	3.71	1.11	.92*	-.54 (.07)	-.47 (.13)
3. My family listened to me	3.55	1.11	.89*	-.40 (.07)	-.48 (.13)
4. I felt that I was an important part of my family	3.70	1.18	.92*	-.60 (.07)	-.49 (.13)
5. My family were supportive	3.82	1.09	.92*	-.65 (.07)	-.23 (.13)
6. The atmosphere at home was encouraging and supportive	3.70	1.16	.93*	-.55 (.07)	-.52 (.13)
7. I felt secure at home	4.04	1.09	.87*	-.96 (.07)	.12 (.13)
8. I knew my parents were looking out for me	4.00	1.08	.91*	-.84 (.07)	-.08 (.13)
9. I was happy at home	3.86	1.08	.89*	-.75 (.07)	-.05 (.13)
10. If times were tough my family helped me feel better	3.72	1.13	.92*	-.57 (.07)	-.44 (.13)
11. My home-life allowed me to feel my life was meaningful	3.72	1.17	.92*	-.62 (.07)	-.45 (.13)
12. My family supported me in reaching my goals	3.73	1.19	.91*	-.64 (.07)	-.46 (.13)

^*^ indicates significance at *p* < .001 level

The fit statistics for all CFA models are illustrated in Table 2. The chi-square statistic was significant for all models, however, this measure is sensitive to large sample size (Tanaka, 1987). All models fitted the data reasonably well, yet the RMSEA was slightly higher than the recommended cut-off for the one-factor model. The BIC indicated that the correlated six-factor model was the best fitting model. All factor loadings were high (**Λ** = 0.89-0.95) and statistically significant at the *p* < 0.001 level (See Table 2). Despite the correlated six-factor model providing the best fit to the data, both the second-order six factor model and the unidimensional model also demonstrated good fit and the factor loadings for these models were all high, positive and statistically significant (See Supplementary Materials 2 and 3).

**Table 2:** Confirmatory Factor Analysis Model Fit Statistics

Model	χ^2^ (df), *p*	TLI	CFI	RMSEA (90% C.I.)	SRMR	BIC
One Factor	547.05 (54), *p* < .001	.950	.959	.081 (.075—.087)	.019	29,441.81
Six Factor First-Order	139.03 (39), *p* < .001	.986	.992	.043 (.035—.051)	.009	28,803.49
Second-Order	267.54 (48), *p* < .001	.975	.982	.057 (.050—.064)	.014	28,968.55

χ^2^ = chi-square test, TLI = Tucker Lewis Index, CFI = Comparative Fit Index, RMSEA = Root Mean Square Error of Approximation, Standardized Root Mean Square Residual, BIC = Bayesian Information Criterion

Total subscale scores were calculated and the range of possible and observed scores was 2 to 10, which suggests that the subscales can also generate sufficient variability in scores. The average scores ranged from 7.25 to 8.04 (*SD* = 2.08–2.27) and internal consistency was excellent (α = 0.98). Subscale level statistics are reported in Supplementary Materials 4 (Table 3).

**Table 3:** Standardized Factor Loadings for Six-Factor CFA Model

	Factors					
Item	Valued	Independent	Support	Secure	Wellness	Growth
I felt my parents valued me	.933	-	-	-	-	-
I felt appreciated by my family	.949	-	-	-	-	-
My family listened to me	-	.897	-	-	-	-
I felt that I was an important part of my family	-	.933	-	-	-	-
My family were supportive	-	-	.917	-	-	-
The atmosphere at home was encouraging and supportive	-	-	.933	-	-	-
I felt secure at home	-	-	-	.901	-	-
I knew my parents were looking out for me	-	-	-	.942	-	-
I was happy at home	-	-	-	-	.888	-
If times were tough my family helped me feel better	-	-	-	-	.921	-
My home-life allowed me to feel my life was meaningful	-	-	-	-	-	.931
My family supported me in reaching my goals	-	-	-	-	-	.916
Factor correlations						
Valued						
Independent	.976					
Support	.958	.970				
Secure	.902	.906	.944			
Wellness	.930	.943	.980	.941		
Growth	.923	.949	.974	.930	.989	

All factor loadings are significant at the *p* < .001 level

Bivariate correlations were calculated for subscales and total MHFS-SF, age, and total scores of depression, anxiety, loneliness, wellbeing, and paranoia. All MHFS-SF subscale scores and total scores were negatively correlated with depression, anxiety, loneliness, and paranoia, and positively correlated with wellbeing and age. All correlations were significant at the *p* < 0.001 level (see Table 4).

**Table 4:** Bivariate correlations for total MHFS-SF scores, age, and mental health outcomes

	Valued Subscale	Independent Subscale	Support Subscale	Secure Subscale	Wellness Subscale	Growth Subscale	MCSF-SF Total
MCSF-SF Total	.94	.94	.96	.93	.95	.95	-
Age	.13	.10	.15	.19	.14	.12	.15
Depression	-.32	-.32	-.31	-.33	-.32	-.34	-.34
Anxiety	-.28	-.28	-.27	-.31	-.29	-.30	-.30
Loneliness	-.29	-.32	-.29	-.29	-.32	-.32	-.32
Wellbeing	.38	.40	.38	.39	.39	.40	.41
Paranoia	-.31	-.32	-.31	-.33	-.31	-.33	-.34

All correlations are significant at the *p* < .001 level

Findings from an independent samples t-test illustrated that mean levels of MHFS-SF scores significantly differed by gender (t (1400) = 1.98, *p* = 0.048), with males (M = 46.0, SD = 12.1) scoring significantly higher than females (M = 44.7, SD = 12.4), although the effect size was very small (*d* = 0.11).

### Multiple Regressions

The first regression model predicted MHFS-SF total scores from demographic variables (age and gender), depression, and anxiety. Findings indicated that depression was negatively associated with MHFS-SF scores (*p* < 0.001); however, age, gender, and anxiety symptoms were not significantly associated with MHFS-SF total scores. Next, a multivariate regression predicting mental health and wellbeing outcomes (loneliness, paranoia and wellbeing) from age, gender, depression, anxiety, and MHSF-SF total scores were conducted. For all outcomes, MHFS-SF scores were significantly associated with the outcome at the *p* < 0.001 level, even after controlling for demographic characteristics and current internalising symptoms. See Table 5 for regression outcomes and standardized coefficients.

**Table 5:** Standardized regression coefficients from multivariate regression predicting mental health and wellbeing outcomes from MHSF-SF total scores

				95% C.I				
Outcome	Variable	*β*	*SE*	Lower	Upper	t	*p*	*R*^*2*^
Loneliness	Intercept	2.758	.141	2.482	3.033	19.60	< .001**	.368
	Age	-.063	.023	-.108	-.019	-2.77	.006*	
	Gender	.035	.045	-.053	.122	.078	.434	
	Depression	.454	.052	.353	.555	8.78	< .001**	
	Anxiety	.077	.053	-.026	.181	1.46	.144	
	MHFS-SF Total	-.131	.025	-.180	-.083	-5.31	< .001**	
Paranoia	Intercept	2.922	.130	2.667	3.176	22.48	< .001**	.410
	Age	-.164	.022	-.207	-.122	-7.57	< .001**	
	Gender	-.038	.042	-.121	.045	-0.89	.373	
	Depression	.161	.046	.071	.251	3.51	< .001**	
	Anxiety	.360	.046	.270	.450	7.85	< .001**	
	MHFS-SF Total	-.148	.023	-.193	-.102	-6.33	< .001**	
Mental Wellbeing	Intercept	2.705	.184	2.345	3.064	14.73	< .001**	.370
	Age	.132	.024	.085	.179	5.49	< .001**	
	Gender	.070	.045	-.018	.157	1.56	.199	
	Depression	-.237	.042	-.319	-.154	-5.63	< .001**	
	Anxiety	-.199	.042	-.280	-.117	-4.79	< .001**	
	MHFS-SF Total	.253	.027	.201	.305	9.51	< .001**	

^*^ indicates significance at *p* < .01 level, ** indicates significance at *p* < .001 level

Finally, bivariate regression models predicting mental health and wellbeing outcomes (loneliness, paranoia, and wellbeing) from age, gender, depression, anxiety, and subscales totals of the MHSF-SF was conducted. For all outcomes, each MHFS-SF subscale was significantly associated with the outcome at the *p* < 0.001 level. See Table 6 for regression outcomes and standardized coefficients.

**Table 6:** Standardized regression coefficients from bivariate regression predicting mental health and wellbeing outcomes from MHFS-SF subscales

				95% C.I				
Outcome	Variable	*β*	*SE*	Lower	Upper	t	*p*	*R*^*2*^
Loneliness	Age	-.244	.024	-.290	-.198	-10.37	< .001***	.060
	Gender	.189	.053	.085	.293	3.56	< .001***	.075
	Depression	.591	.021	.549	.632	28.08	< .001***	.349
	Anxiety	.540	.023	.495	.585	23.69	< .001***	.292
	Valued Subscale	-.289	.027	-.342	-.236	-10.67	< .001***	.084
	Independent Subscale	-.315	.027	-.367	-.262	-11.73	< .001***	.099
	Support Subscale	-.293	.027	-.346	-.240	-10.83	< .001***	.086
	Secure Subscale	-.287	.028	-.342	-.232	-10.26	< .001***	.082
	Wellness Subscale	-.319	.027	-.372	-.266	-11.74	< .001***	.102
	Growth Subscale	-.322	.027	-.375	-.269	-11.83	< .001***	.104
Paranoia	Age	-.336	.022	-.380	-.292	-15.00	< .001***	.113
	Gender	.169	.053	.065	.274	3.19	.001**	.111
	Depression	.577	.020	.537	.617	28.30	< .001***	.332
	Anxiety	.595	.020	.556	.634	30.06	< .001***	.354
	Valued Subscale	-.306	.025	-.356	-.256	-12.05	< .001***	.094
	Independent Subscale	-.317	.025	-.367	-.267	-12.48	< .001***	.100
	Support Subscale	-.313	.026	-.363	-.263	-12.22	< .001***	.098
	Secure Subscale	-.334	.025	-.384	-.284	-13.13	< .001***	.112
	Wellness Subscale	-.306	.026	-.356	-.256	-11.96	< .001***	.094
	Growth Subscale	-.329	.026	-.379	-.279	-12.90	< .001***	.108
Mental Wellbeing	Age	.291	.023	.245	.337	12.39	< .001***	.085
	Gender	-.113	.053	-.217	-.008	-2.11	.035*	.291
	Depression	-.537	.025	-.586	-.487	-21.34	< .001***	.288
	Anxiety	-.521	.025	-.570	-.472	-20.79	< .001***	.271
	Valued Subscale	.383	.027	.331	.435	14.42	< .001***	.147
	Independent Subscale	.398	.027	.346	.450	14.94	< .001***	.158
	Support Subscale	.380	.027	.328	.432	14.31	< .001***	.144
	Secure Subscale	.389	.027	.337	.442	14.60	< .001***	.152
	Wellness Subscale	.389	.026	.337	.441	14.77	< .001***	.151
	Growth Subscale	.395	.026	.344	.447	15.07	< .001***	.156

^*^ indicates significance at *p* < .05 level, ** indicates significance at *p* < .01 level, *** indicates significance at the *p* < .001 level

## Discussion

The primary objective of the present study was to develop and validate the MHFS-SF, a short version of the Memories of Home and Family Scale (MHFS; Shevlin et al., 2022). The MHFS-SF includes twelve items which capture memories of positive emotions and experiences pertaining to being a valued member of the family, an independent member of the family, feeling supported within family, feeling secure within family, having a sense of wellbeing at home, and opportunities for growth and meaning.

Aligning with the original hypothesis, CFA results supported the representation of the MHFS-SF as a multidimensional measure as it was in the original version of the scale (Shevlin et al., 2022). Each of the subscales were found to be strongly (0.83—0.96) and significantly (*p* < 0.001) correlated with one another, while all items were found to load strongly (0.89—0.94) and significantly (*p* < 0.001) onto their respective subscales. This suggests that individual sub-scale scores can be computed for the MHFS-SF. Notably, the unidimensional and second-order models were also considered as appropriate structural representations of the MHFS-SF in the present study, supporting the idea that the MHFS-SF can be used to generate both total and sub-scale scores. Findings from the present study indicate that the MHFS-SF retains the psychometric soundness of the original scale. The internal consistency of the MHFS-SF items as well as the MHFS-SF subscales was commensurate with that of the original scale (Shevlin et al., 2022). Hence, the reduction to a 12-item scale has not compromised the internal consistency of the scale itself. Moreover, as anticipated, the MHFS-SF demonstrated high levels of convergent and discriminant validity. Specifically, total MHFS-SF scores were significantly and negatively correlated with depression (*r* = -0.34), anxiety (*r* = -0.30), paranoia (r = -0.34), and loneliness (*r* = -0.32); and positively associated with wellbeing (*r* = 0.41). These findings are mostly consistent with the initial validation study where total MHFS scores were significantly and negatively correlated with depression (*r* = -0.35), anxiety (*r* = -0.27), and loneliness (*r* = -0.53); and positively associated with resilience (*r* = 0.26). The magnitude of the correlations between the individual subscales and loneliness, depression, anxiety, paranoia, and wellbeing were largely similar to those seen for the total scores, indicating that the individual subscales are independently associated with mental health outcomes in the anticipated direction. Collectively, these findings support the convergent and discriminant validity of the MHFS-SF total and sub-scale scores.

The final aims of the present study were to examine predictors of both total and sub-scale MHFS-SF scores and the associations between total and sub-scale MHFS-SF scores and mental health outcomes. Consistent with prior research (Almeida et al., 2021), older age was identified as a significant positive predictor of total and subscale MHFS-SF scores. Regarding mental health outcomes, findings from the present study illustrated how MHFS-SF total scores were significant negative predictors of loneliness (β = -0.13), paranoia (β = -0.15), and loneliness (β = -0.21), and a significant positive predictor of wellbeing (β = 0.25). These associations held after accounting for age, gender, and current internalising symptoms. Research has shown how emotional states can influence adult reporting of early life experiences (Colman et al., 2016), and hence statistically adjusting for current internalising symptoms accounts for this to a degree. The significant association between MHFS-SF total and sub-scale scores and paranoia symptoms, a non-internalising mental health problem, indicates that MHFS-SF scores are associated with mental health outcomes regardless of current mental health states. Similar patterns of association between individual subscales and mental health outcomes were also found, indicating that the various facets of positive experiences in the home environment can predict mental health to a similar extent as the overall score. Moreover, these findings reaffirm the beneficial influence of positive early life experiences on mental health (e.g., Crandall et al., 2019; Doom et al., 2021; Narayan et al., 2018).

Findings from this study should be considered in light of several limitations. First, the data pertaining to the MHFS-SF was collected at one time-point and hence, the temporal stability of scores could not be assessed. Second, the present study included participants from a relatively affluent western country, and hence, the generalizability of the findings to other countries and cultural contexts warrants exploration. Similarly, given that much of the sample reported high scores on the MHFS-SF, the utility of the scale must be examined in samples where there is likely to be individuals who are not from cohesive or supportive families. Third, the MHFS-SF was reported retrospectively, and hence, it is possible that there were some biases in reporting including false negatives and measurement error (Hardt & Rutter, 2004) as well as current emotional states influencing responding (Colman et al., 2016). That being said, there is evidence to suggest that differences between retrospective and prospective reporting are minimal in nature (Pattern et al., 2015). Fourth, given that the MHFS-SF items largely revolve around the family unit, it is possible that some respondents may answer items with respect to a particular family member. As an example, for the MHFS-SF item *‘My family were supportive’*, it is possible that despite family members being supportive in general that an individual may respond to this item with respect to one unsupportive family member. Fifth, although the present study examines the validity and reliability of the MHFS-SF, further research is required to determine whether the reduction in items from the original scale eases burden and improves the utility of the scale. Finally, it was not possible to examine the concurrent validity of the MHFS-SF, and hence, future studies may wish to examine the association between the MHFS-SF and other independent measures of BCEs (e.g., BCE scale).

In summary, the current study described the development and initial validation of the short version of the MHFS using a large representative sample of adults from the UK general population. The MHFS-SF maintains the reliability, validity, and structure of the original MHFS while aiming to improve its brevity and ease of application. We believe that the MHFS-SF will be useful in research as well as in clinical practice-based settings where the scale can be used to identify individuals lacking in such experiences or to draw on these experiences as a potential interventive strategy. The MHFS-SF has the advantage of allowing for the calculation of both total and sub-scale scores; the former may be optimal for getting a broad picture of all of the positive experiences at home with family, and the latter may be best for evaluating specific elements of such experiences. The clinical utility of these experiences for those undergoing psychotherapy for various mental health issues should also be explored in further research. Finally, the interplay between positive and negative experiences for symptom expression across different developmental stages requires further research. The MHFS-SF is now freely available in the public domain (see Supplementary Materials 5). Further research is required to assess the temporal stability of the MHFS-SF as well as to validate the MHFS-SF across different populations including clinical samples where such experiences may be less plentiful and across different countries and cultural contexts.

## Supplementary Information

Below is the link to the electronic supplementary material.

Supplementary file1 (DOCX 27 KB)
