# Supplementary material for: Development and initial validation of a short form of the Memories of Home and Family Scale
Source: Advers Resil Sci. 2023 Apr 29:1–10. Online ahead of print. doi: 10.1007/s42844-023-00097-x (PMC10148702; doi:10.1007/s42844-023-00097-x)
Supplement: Supplementary file 1 — Supplementary file1 (DOCX 27 KB) [file 42844_2023_97_MOESM1_ESM.docx]

**Supplementary Materials 1:** Descriptive Statistics for Mental Health and Wellbeing Scales

| **Scale** | **Mean** | ***SD*** | **Min** | **Max** | **Skew(SE)** | **Kurtosis (SE)** |
| --- | --- | --- | --- | --- | --- | --- |
| Depression | 5.12 | 6.23 | 0 | 27 | 1.46 (.07) | 1.60 (.13) |
| Anxiety | 4.21 | 5.48 | 0 | 21 | 1.37 (.07) | 1.01 (.13) |
| Loneliness | 4.78 | 1.90 | 0 | 9 | .72 (.07) | -.56 (.13) |
| Paranoia | 11.50 | 5.15 | 0 | 25 | .37 (.07) | -.76 (.13) |
| Mental Wellbeing | 23.5 | 6.28 | 0 | 25 | -.51 (.07 | .15 (.13) |

**Supplementary Materials 2:** Standardized Factor Loadings for Second-Order CFA Model

|  | **Factors** | | | | | |
| --- | --- | --- | --- | --- | --- | --- |
| **Item** | **Valued** | **Independent** | **Support** | **Secure** | **Wellness** | **Growth** |
| I felt my parents valued me | .934 | - | - | - | - | - |
| I felt appreciated by my family | .947 | - | - | - | - | - |
| My family listened to me | - | .894 | - | - | - | - |
| I felt that I was an important part of my family | - | .935 | - | - | - | - |
| My family were supportive | - | - | .917 | - | - | - |
| The atmosphere at home was encouraging and supportive | - | - | .933 | - | - | - |
| I felt secure at home | - | - | - | .900 | - | - |
| I knew my parents were looking out for me | - | - | - | .942 | - | - |
| I was happy at home | - | - | - | - | .886 | - |
| If times were tough my family helped me feel better | - | - | - | - | .923 | - |
| My home-life allowed me to feel my life was meaningful | - | - | - | - | - | .928 |
| My family supported me in reaching my goals | - | - | - | - | - | .918 |

Note: All factor loadings were significant at the *p* < .001 level.

**Supplementary Materials 3:** Standardized Factor Loadings for Unidimensional CFA Models

| **Item** | **CFA** | **CFA with MI** |
| --- | --- | --- |
| 1. I felt my parents valued me | .904 | .896 |
| 1. I felt appreciated by my family | .916 | .909 |
| 1. My family listened to me | .875 | .875 |
| 1. I felt that I was an important part of my family | .915 | .914 |
| 1. My family were supportive | .912 | .913 |
| 1. The atmosphere at home was encouraging and supportive | .927 | .928 |
| 1. I felt secure at home | .856 | .850 |
| 1. I knew my parents were looking out for me | .894 | .888 |
| 1. I was happy at home | .873 | .874 |
| 1. If times were tough my family helped me feel better | .907 | .910 |
| 1. My home-life allowed me to feel my life was meaningful | .911 | .914 |
| 1. My family supported me in reaching my goals | .901 | .904 |

Note: All factor loadings were significant at the *p* < .001 level

**Supplementary Materials 4:** Subscale-Level Descriptive Statistics for MHFS-SF

| **Subscale** | **Mean** | ***SD*** | **Min** | **Max** | **Skew(SE)** | **Kurtosis (SE)** |
| --- | --- | --- | --- | --- | --- | --- |
| Valued | 7.45 | 2.15 | 2 | 10 | -.53 (.07) | -.44 (.13) |
| Independent | 7.25 | 2.19 | 2 | 10 | -.53 (.07) | -.42 (.13) |
| Support | 7.52 | 2.16 | 2 | 10 | -.62 (.07) | -.30 (.13) |
| Secure | 8.04 | 2.08 | 2 | 10 | -.90 (.07) | .07 (.13) |
| Wellness | 7.58 | 2.11 | 2 | 10 | -.68 (.07) | -.19 (.13) |
| Growth & Meaning | 7.45 | 2.27 | 2 | 10 | -.63 (.07) | -.43 (.13) |

**Supplementary Materials 5**

**Memories of Home and Family During Childhood Scale- Abbreviated**

The following questions were designed to explore your memories of your childhood at home and with your family. The following questions are about how you recall your early life up to the age of 16 years. Please complete the scale by circling the most appropriate number under each statement. Up to the age of 16 years………

| Up to the age of 16 years……… | Never | Very Rarely | Sometimes | Frequently | Always |
| --- | --- | --- | --- | --- | --- |
| 1. I felt my parents valued me | 1 | 2 | 3 | 4 | 5 |
| 1. I felt appreciated by my family | 1 | 2 | 3 | 4 | 5 |
| 1. My family listened to me | 1 | 2 | 3 | 4 | 5 |
| 1. I felt that I was an important part of my family | 1 | 2 | 3 | 4 | 5 |
| 1. My family were supportive | 1 | 2 | 3 | 4 | 5 |
| 1. The atmosphere at home was encouraging and supportive | 1 | 2 | 3 | 4 | 5 |
| 1. I felt secure at home | 1 | 2 | 3 | 4 | 5 |
| 1. I knew my parents were looking out for me | 1 | 2 | 3 | 4 | 5 |
| 1. I was happy at home | 1 | 2 | 3 | 4 | 5 |
| 1. If times were tough my family helped me feel better | 1 | 2 | 3 | 4 | 5 |
| 1. My home-life allowed me to feel my life was meaningful | 1 | 2 | 3 | 4 | 5 |
| 1. My family supported me in reaching my goals | 1 | 2 | 3 | 4 | 5 |
